# Scrutiny of genome‐wide somatic mutation profiles in centenarians identifies the key genomic regions for human longevity

**DOI:** 10.1111/acel.13916

**Published:** 2023-07-03

**Authors:** Hao‐Tian Wang, Long Zhao, Li‐Qin Yang, Ming‐Xia Ge, Xing‐Li Yang, Zong‐Liang Gao, Yu‐Peng Cun, Fu‐Hui Xiao, Qing‐Peng Kong

**Affiliations:** ^1^ State Key Laboratory of Genetic Resources and Evolution, Key Laboratory of Healthy Aging Research of Yunnan Province, Kunming Key Laboratory of Healthy Aging Study, KIZ/CUHK Joint Laboratory of Bioresources and Molecular Research in Common Diseases Kunming Institute of Zoology, Chinese Academy of Sciences Kunming China; ^2^ Kunming College of Life Science University of Chinese Academy of Sciences Kunming China; ^3^ Pediatric Research Institute/Ministry of Education Key Laboratory of Child Development and Disorders/National Clinical Research Center for Child Health and Disorders Children's Hospital of Chongqing Medical University Chongqing China; ^4^ CAS Center for Excellence in Animal Evolution and Genetics Chinese Academy of Sciences Kunming China

**Keywords:** centenarian, healthy aging, longevity, somatic mutation

## Abstract

Somatic mutations accumulate with age and are associated closely with human health, their characterization in longevity cohorts remains largely unknown. Here, by analyzing whole genome somatic mutation profiles in 73 centenarians and 51 younger controls in China, we found that centenarian genomes are characterized by a markedly skewed distribution of somatic mutations, with many genomic regions being specifically conserved but displaying a high function potential. This, together with the observed more efficient DNA repair ability in the long‐lived individuals, supports the existence of key genomic regions for human survival during aging, with their integrity being of essential to human longevity.

## Graphical abstract

By systematically analyzing the somatic mutation landscapes of 73 centenarians and 51 controls using whole genome sequencing, Wang et al. identified a number of genomic regions (viz., CSM‐FRs) that are free of any somatic mutations in the centenarians but are always mutated in the general older people. Importantly, the high conservation and function potential of these genomic regions indicate the necessity of their integrity for human longevity.

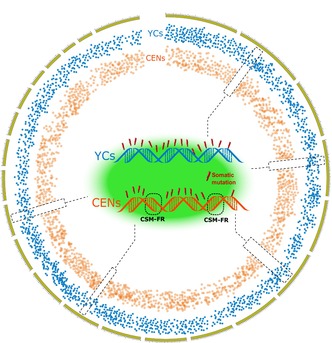

**Abbreviations**
CEN: centenarian
CSM‐FR: centenarian somatic mutation‐free regions
CSM‐CR: centenarian somatic mutation cluster regions
DRH: DNA repair hotspots
DSM: disease susceptibility mutation
SSBS: somatic single‐base substitution
VAF: variant allele frequency
WGS: whole genome sequencing
YC: younger controls

## 1 INTRODUCTION, RESULTS AND DISCUSSION

Centenarians (CENs) keep relatively healthy, as shown by epidemiological surveys, over their remarkable extraordinarily long lives, suggesting an ability to suppress certain serious age‐related diseases, such as atherosclerosis, coronary artery disease, and Alzheimer's disease (AD) (Borras et al., 2020; Evert et al., 2003; Newman & Murabito, 2013). However, the genetic basis underlying this survival advantage is largely unknown. Here, we attempted to address this issue by generated and analyzed genome‐wide somatic mutation profiles of CENs and younger controls (YCs) due to the following reasons. First, somatic mutation accumulation, an age‐associated and interspecies‐conserved event (Dollé et al., 2000; Vijg, 2021; Zhang et al., 2019), results from erroneous DNA replication or repair and is suggested to be an important driving factor for aging and age‐related diseases (Heimlich & Bick, 2022; Heyde et al., 2021; Vijg & Dong, 2020). Second, somatic mutation rates show a strong inverse relationship with life spans across species (Cagan et al., 2022). Third, only a small number of longevity‐related germ line variants have been identified in the longevity cohorts using genome‐wide scanning method (Deelen et al., 2019), although emerging evidence suggests that CENs may be at greater threat from genome‐wide somatic mutations due to their much longer life spans (Holstege et al., 2014; Zhang et al., 2019). Given that the somatic mutations occur nonrandomly in the genome (Martincorena et al., 2012), we hypothesize that CENs may escape certain harmful mutational events in genomic regions closely related to human health and survival.

In the current study, we screened for somatic mutations based on whole genome sequencing of peripheral blood samples from 73 female CENs (age: 100.5 ± 3.7 years) and 51 gender‐matched YCs (age: 60.3 ± 7.8 years). A total of 110,157 somatic single‐base substitution (SSBS) mutations were identified in the 124 individuals (Table S1). We observed a high degree of consistency in the spatial distribution and mutational spectra of the somatic mutations identified in our study and those obtained by error‐corrected sequencing methods (Abascal et al., 2021; Xing et al., 2021) (Table S5, Figure S1a–d). No difference in the mutation calling sensitivity was found between the two groups (Figure S1h).

**FIGURE 1:**
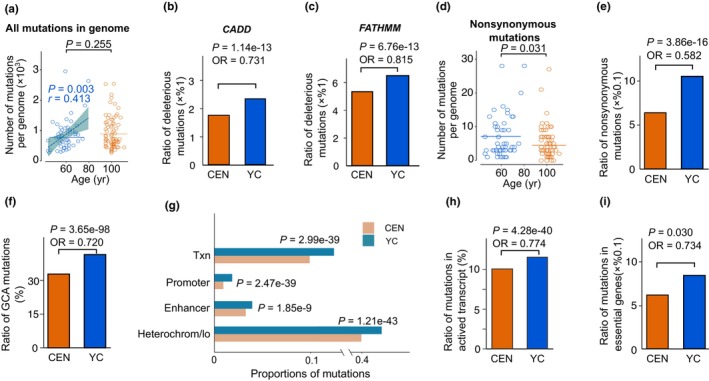
CEN mutation severities were lower than YC mutation. (a) Mutational burdens of CENs and YCs. Two‐sample tests were performed by Wilcoxon rank‐sum test, and horizontal lines represent means. Dashed lines represent fitted lines for YC mutations. Correlations were analyzed using Pearson correlation tests. Blue represents YCs and orange represents CENs. (b,c) Ratios of deleterious mutations in CENs and YCs, predicted by CADD (b) and FATHMM (c). (d) Nonsynonymous mutation numbers in CENs and YCs. Two‐sample tests were performed by Wilcoxon rank‐sum test, and horizontal lines represent means. (e) Proportions of nonsynonymous mutations in CENs and YCs. (f) Proportions of mutations in different genomic subcompartments in CENs and YCs. *p* values were calculated by Fisher's exact test. (g) Distributions of mutations in ChromHMM segments. *p* values were calculated by Fisher's exact test. “Txn,” transcription (h) Proportions of mutations located in active/inactive transcriptional regions in CENs and YCs. *p* values were calculated by Fisher's exact test. (i) Proportions of mutations located in essential/nonessential genes in CENs and YCs. *p* values were calculated by Fisher's exact test.

Overall, CENs contained slightly more mutations compared to YCs (950 vs. 881 per sample, on average; Figure 1a). A positive correlation between mutation number and age was observed in YC samples (Figure 1a); however, variant allele frequency (VAF) was, on average, lower in CENs relative to YCs (Figure S1f), suggesting that cells carried fewer somatic mutations in CENs than expected. We also estimated the harmfulness of each SSBS using combined annotation‐dependent depletion (CADD) (Rentzsch et al., 2019) and functional analysis through hidden Markov models (FATHMM) (Shihab et al., 2013) and found that the ratio of predicted deleterious SSBSs was significantly smaller in CENs than in YCs (Figure 1b,c, Figure S1g).

We then determined distribution bias in somatic mutations across the genome between CENs and YCs, with CENs carrying fewer SSBSs in functional elements. For example, the number of exonic SSBSs per sample was lower in CENs compared to YCs (~7 per CEN vs. ~9 per YC, Figure S2a). The exonic SSBS ratio was also significantly lower in CENs than in YCs, but increased with age in YCs (Figure S2). Likewise, the number and ratio of nonsynonymous SSBSs (Figure 1d,e) were both significantly lower in CENs compared with YCs. The CENs also carried significantly fewer SSBSs in the gene‐enriched genomic compartment (i.e., genome compartment A, GCA), transcription regulation‐associated regions (e.g., transcribed regions, promoters, and enhancers), and transcriptionally active regions (Figure 1f–h, Figure S3a). Similar results were also observed in human essential genes (Figure 1i). As most (99.18%) identified SSBSs were nonexonic, we investigated the distribution of SSBSs in the predicted regulatory regions (classified into five categories: H3K4me3, H3K27ac, ENCODE cCRE, oRegAnno, and Ensembl regulatory elements), and found that CENs contained fewer SSBSs in these regions than YCs (Figure S3b).

**FIGURE 2:**
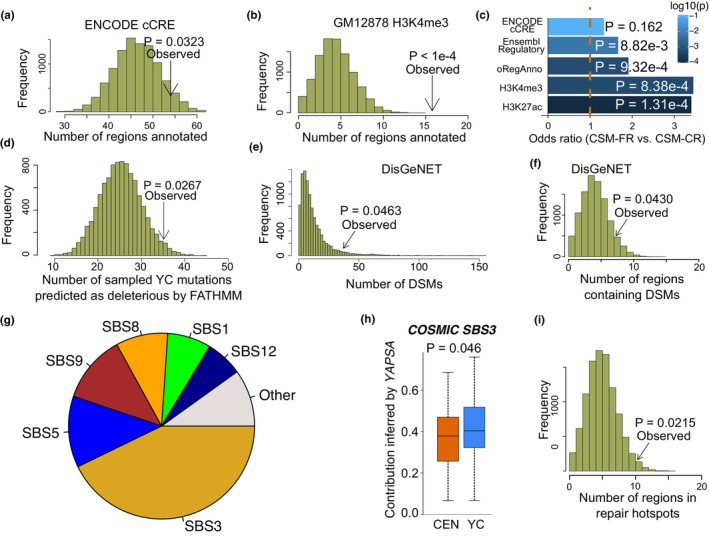
CSM‐FRs were preferentially located in key genomic regions. (a,b) Distribution of randomly sampled regions located in regulatory elements defined by ENCODE cCRE data archive (a) and corresponding distribution of H3K4me3 (b). Arrows represent the observed numbers of the CSM‐FRs located in the corresponding regulatory elements. (c) CSM‐FRs were preferentially located in regulatory elements compared to CSM‐CRs. *p* values were calculated by Fisher's exact test. (d) Frequency distribution of randomly sampled YC mutations predicted as deleterious. Arrow represents observed number of deleterious YC mutations located in CSM‐FRs. (e) Frequency distribution of DSMs located in randomly selected regions. Arrow represents observed number of DSMs located in CSM‐FRs. (f) Frequency distribution of randomly sampled regions containing DSMs. Arrow represents observed number of DSM‐containing CSM‐FRs. (g) The overall contributions of mutational signatures among all 124 samples. (h) Contribution of COSMIC SBS3 identified by YAPSA. Two‐sample tests were performed by Wilcoxon rank‐sum test. (i) Frequency distribution of randomly sampled regions located in DRHs. Arrow represents the observed number of the CSM‐FRs located in DRHs.

Interestingly, we identified 137 genomic regions (average width: 3587 bp) prone to be mutated in YCs but extremely conserved in CENs (hereafter defined as CEN somatic mutation‐free regions, CSM‐FRs) (Figures S4a and S5a, Table S2; see Methods). No read‐coverage difference in the CSM‐FRs was observed between the CEN and YC groups (Figure S5e). Further analysis revealed that these CSM‐FRs were not randomly distributed (Figure S5b,c), but were significantly overrepresented in the five regulatory regions compared to randomly sampled regions across the genome (Figure 2a,b, Figure S6). Importantly, the CSM‐FRs were significantly overrepresented in four of the five regulatory regions compared to CEN somatic mutation cluster regions (CSM‐CRs; see Methods), except for ENCODE cCRE (Figure 2c).

The above results suggest that the CSM‐FRs, or at least some of them, may be so vital that any mutagenesis therein would be highly deleterious. We then speculated that any observed somatic mutations in the CSM‐FRs may be more harmful or, alternatively, the identified pathogenic mutations may have much higher possibility to occur in CSM‐FRs than any other genomic regions. Indeed, by studying YC mutations located in the CSM‐FRs, we found that these mutations were more likely to be deleterious than those outside the CSM‐FRs (Figure 2d). Furthermore, based on disease susceptibility mutations (DSMs) from the DisGeNET database (Piñero et al., 2021), we found that CSM‐FRs preferentially covered more DSMs than random genomic regions and CSM‐CRs (Figure 2e, Figure S7). The observed proportion of CSM‐FRs containing DSMs was also significantly higher than that in any other random genomic regions and CSM‐CRs (Figure 2f, Figure S8). Interestingly, enrichment analysis showed that genes in CSM‐FRs were significantly enriched in the cyanate metabolic pathway and cyanate catabolic process (*p* < 0.05; Table S3), the dysregulation of which may irreversibly disrupt lipoprotein function and contribute to cardiovascular disease (CVD) (Gorisse et al., 2016; Wang et al., 2007), echoing the decreased incidence of CVD in CENs (Borras et al., 2020).

To investigate the factors underlying CEN‐specific SSBS distribution, we performed mutational signature analysis of samples using the *YAPSA* method (Hübschmann et al., 2021) (Figure 2g; Figure S9a). In accordance with previous studies (Alexandrov et al., 2020; Zhang et al., 2019), two age‐related clocklike signatures, SBS5 and SBS40 in Catalogue of Somatic Mutations in Cancer (COSMIC), were also more prevalent in CEN group (Figure S9b,c). Results showed that the proportion of SBS3, which is associated with failure in DNA homologous recombination repair (HRR) (Alexandrov et al., 2015), was significantly lower in CENs (Figure 2h, Figure S9d). In addition, signatures SBS6 and SBS15, ascribed to defective DNA repair, were also less common in CENs (Table S4). These findings suggest that CSM‐FRs may arise from less error‐prone DNA repair machinery in CENs and are likely to be DNA repair hotspots (DRHs), as described recently (Reid et al., 2021). As expected, the CSM‐FRs were preferentially located in DRHs (Wu et al., 2021) (Figure 2i). In addition, we also found that CENs exhibited a higher activity of postreplication repair pathway, which was evaluated using the GSVA method based on the transcriptome data of the same batch of samples (Xiao et al., 2018, 2022) (Figure S9e). Accordingly, these findings suggested that this CEN‐specific pattern of somatic mutations may be partially explained by a more efficient DNA repair ability, which is likely to be a conserved mechanism associated with longevity and healthy aging in various species, including humans (Garagnani et al., 2021; Kowalczyk et al., 2020; Zhang et al., 2021).

In short, by analyzing genome‐wide somatic mutation profiles in a cohort of centenarians and younger controls, our study provides evidence showing that centenarians are characterized by the presence of a number of somatic mutation‐free regions, which are specifically conserved in CENs but have high potential for human survival and health. Further analysis suggested these regions (viz., CSM‐FRs) were enriched with gene regulatory regions, and maintaining their integrity during aging likely via a centenarian‐specific elevated DNA repair ability. Since our samples (centenarians and younger controls) are all females, whether the currently observed pattern in somatic mutation is confined to female or also applied to male still awaits further investigation. Although previous study has shown that identifying blood somatic mutation using bulk high‐throughput sequencing is comparable to that based on single‐cell derived colonies (Yadav et al., 2016), the estimated mutational burden by bulk sequencing varies widely between studies (Holstege et al., 2014; Pich et al., 2022; Van Den Akker et al., 2021; Yadav et al., 2016), suggesting that somatic mutation burden may be (hyper‐)variable among different individuals or, alternatively, more efforts shall be paid on improving the accuracy in somatic mutation calling. Future studies utilizing high‐precision methods (e.g., error‐corrected duplex sequencing and sequencing on single‐cell derived colonies) may be of help to offer a more refined insight of the roles of mutagenesis in healthy aging. Nonetheless, this observed markedly skewed distribution of somatic mutations across CEN genomes offers a new explanation for the genomic basis for the considerable survival advantage of these long‐lived individuals at the mutagenic level.

## 2 METHODS

### 2.1 Subjects, sampling, and sequencing

We collected peripheral whole blood samples from 124 female participants, including 73 centenarians (CENs) and 51 younger controls (YCs). All controls were F1 spouses of CENs. All participants provided signed informed consent regarding participation and experimental goals and protocols. The study proposal was approved by the Ethics Committee at Kunming Institute of Zoology, Chinese Academy of Sciences. Genomic DNA was extracted using a AxyPrep Blood Genomic DNA Maxiprep Kit (Corning, USA) and extracted DNA quality was assessed using a Qubit dsDNA High Sensitivity Assay (ThermoFisher Scientific, USA). Genomic DNA was fragmentized into 100–300 bp fragments using the Covaris S2 system (Covaris Inc., USA). A whole genome sequencing (WGS) library was prepared using a TruSeq DNA PCR‐Free Kit (Illumina, USA). Final qualified libraries were sequenced on the HiSeq NovaSeq 6000 platform (Illumina, USA) by Biolinker Technology Co., Ltd. (Kunming, China). The samples were sequenced to a mean depth of 37.1× (~111.4 Gbp per sample).

### 2.2 Mapping and somatic mutation calling

Raw fastq files were trimmed using fqtools_plus v1.0.0 with default parameters. The trimmed fastq files were then mapped on the human reference genome GRCh38 using BWA v0.7.17 with default parameters. The mapping‐generated bam files were then preprocessed (i.e., sorted, fixed, deduplicated, and indexed) using SAMtools v1.13 and Genome Analysis Toolkit (GATK) v4.0.8. The base sequencing quality scores of the bam files were then modified using the BaseRecalibrator program in GATK. To accelerate analysis, the scatter–gather strategy was used to split the bam files into 20 files for simultaneous preprocessing. Somatic mutation calling was performed using the MuTect2 program in tumor‐only mode, which was widely adapted in recent studies for somatic mutation identification in the absence of paired control samples (Sun et al., 2021; Wilkinson et al., 2020). Variants identified using the 1000 Genome Project were used as panels of normal (PoN). Variant allele frequency (VAF) was calculated as the ratio of mutant to all mapped reads of each somatic mutation. The mutation calling pipeline is available at https://github.com/ritianjiang/CSMFR‐identify.

### 2.3 Somatic mutation filtering

We referred to the somatic mutation filtering pipeline that adapted in a recent longevity study (Garagnani et al., 2021) and made some modifications. The retained SSBS mutations satisfied the following criteria: (1) “Pass” FILTER flag; (2) Located in autosomes and X chromosome; (3) Not covered in hg38 genomic SuperDups file from University of California Santa Cruz (UCSC) genome browser; (4) Alternative allele frequency less than 0.5; (5) Depth not less than 20 and not more than 120; (6) Number of reads supporting alternative allele not less than 3; and (7) Minor allele frequency <1% in the Genome Aggregation Database (gnomAD) v3.0 project (Karczewski et al., 2020). (8) Observed less than twice in the cohort. We then adopted the FreeBayes v1.3.6 with “pooled caller” mode to recall the somatic mutations. In total, 110,157 mutations identified by both MuTect2 and FreeBayes were used in subsequent analyses.

### 2.4 Estimation of mutation calling quality

To evaluate the quality of our mutation calling results, a read coverage‐based method (Starks et al., 2021) was used to compare the mutation calling sensitivities between CEN and YC, and no significant sensitivity difference was observed (Figure S1h). We used GenometriCorr R package to compare the spatial distributions of mutations from middle‐aged donors (i.e., 59–63 years old) between our data and that utilizing the error‐corrected sequencing (Abascal et al., 2021). We then extracted the mutational spectra of our dataset and found them similar to those from a recent peripheral blood mononuclear cell (PBMC) dataset generated by multiplexed end‐tagging amplification of complementary strands (META‐CS) (Xing et al., 2021) (Figure S1a–d; Table S6). We also performed clonality inference by PyClone2 (Roth et al., 2014) and observed no significant clonality difference between the CEN and YC groups (Figure S1e). Among the 124 samples, six harboring clonal hematopoiesis (CH) mutations (Bick et al., 2020) were determined as potential CH carriers (Table S7).

### 2.5 Identification of CSM‐FRs

We clustered the 110,157 mutations by genomic location. Among the 3285 mutation clusters obtained, those containing mutations merely from YCs (viz., indicating the corresponding genomic region to be (hyper‐)variable in YCs but highly conserved in CENs) were defined as CSM‐FRs. Clusters containing only CEN mutations were defined as CSM‐CR. In total, we identified 137 CSM‐FRs and 517 CSM‐CRs.

Mutation cluster was defined as three or more mutations whose mutual distances were closer than 5 kb. The distance threshold (i.e., <5 kb) was referred to Zhang et al (Zhang et al., 2019). The mutation number threshold (i.e., > = 3) was chosen due to there was only 1.82% (32/1762) CSM‐FRs containing more than three mutations (Figure S4b), this cutoff can reduce the false positive rate compared to a cutoff of two mutations. Besides, we also identified the CSM‐FRs by setting distance threshold as 3 kb, 4 kb, 6 kb, 8 kb, and 10 kb. The recall rates of the thresholds settings were 61.3%, 80.3%, 97.0%, 84.7%, and 75.2% (Figure S4c). There is no significant difference in average coverage between CENs and YCs in CSM‐FRs (Figure S5e). These results showed that the identified CSM‐FRs were robust.

To test whether our identified CSM‐FRs were biased by clonal hematopoiesis (CH), we removed the six potential CH carriers and found that the vast majority (97.67%) of CSM‐FRs persisted, indicating that the identification result is less influenced by clonality. To determine whether some individuals with extreme mutation patterns may have influence on the CSM‐FR identification, we subsampled the individuals by leave‐one‐out method and found that the average CSM‐FR recall ratio was 98.11% (Figure S5d).

### 2.6 Mutation annotation

We used the Ensembl (104) Variant Effect Predictor (VEP) to annotate mutations in genomic regions. The annotated vcf file was converted into maf format. We used CADD and FATHMM to evaluate the damage effect of each mutation. Mutations with a PHRED‐formatted score > 15 were defined as “damaging” based on CADD annotation. The FATHMM‐MKL program was used to determine damaging/nondamaging status. Gene essentiality data were retrieved from the OGEE v2 database (Chen et al., 2017), and genes with contradictory evidence were discarded. Known associations between variants and phenotype were retrieved from the DisGeNET database. We only retained variants linked to disease. Functional enrichment analysis for CSM‐FRs was perform using g:Profiler (https://biit.cs.ut.ee/gprofiler) with default settings. DNA repair hotspots (DRHs) identified by SAR‐seq were obtained from the NCBI Gene Expression Omnibus database (GEO, GSE167257).

### 2.7 Mutational signature analysis and DNA repairment analysis

Reference SSBS signatures were obtained from COSMIC Mutational Signatures. We extracted and visualized the proportions of identified SSBS signatures using the R package YAPSA. We used the R package deconstructSigs (Rosenthal et al., 2016) to extract SSBS signatures to verify the results. The RNA‐seq data of the 124 samples were from our previously published papers (Xiao et al., 2018, 2022). The transcripts per million (TPM) was estimated using Salmon‐1.1.0. To analyze individual pathway‐level activities in each sample, the pathway‐level analysis of gene expression was performed using “plage” function in GSVA R package v1.40.1 (Tomfohr et al., 2005).

### 2.8 Statistical analysis

We used R v4.1.0 to perform all statistical analyses, including Fisher's exact test and Wilcoxon rank‐sum test.

## AUTHOR CONTRIBUTIONS

Q.‐P.K. conceived the project. Q.‐P.K., F.‐H.X., and Y.‐P.C. supervised the project. H.‐T.W., F.‐H.X., and Q.‐P.K. wrote the manuscript. H.‐T.W., L.Z., L.‐Q.Y., M.‐X.G., X.‐L.Y., Z.‐L.G., and F.‐H.X. collected the samples. H.‐T.W., L.Z., and Z.‐L.G. performed the experiments. H.‐T.W., F.‐H.X., and Y.‐P.C analyzed the data. All authors reviewed and approved the manuscript.

## FUNDING INFORMATION

This work was supported by grants from the National Key R&D Program of China (No. 2018YFE0203700), CAS Project for Young Scientists in Basic Research (YSBR‐076), West Light Foundation (to F.‐H.X.) of the Chinese Academy of Sciences, National Natural Science Foundation of China (82071595), Yunnan Fundamental Research Projects (202101AS070058, 202201AS070080, 202301AT070281), High‐level Talent Promotion and Training Project of Kunming (Spring City Plan; 2020SCP001), Yunling Scholar of Yunnan Province, and the Reserve Talent Project of Young and Middle‐aged Academic and Technical Leaders in Yunnan Province (202305AC160029).

## CONFLICT OF INTEREST STATEMENT

The authors declare no competing interests.

## Supporting information


Tables S1–S7

Figures S1–S9

## Data Availability

The R code used to generate results in this study is freely available at https://github.com/ritianjiang/CSMFR‐identify. The original data and scripts for generating the figures are available upon request. All raw sequence data in this paper have been deposited in the Genome Sequence Archive (GSA) under accession number HRA002853.
