# Supplementary material for: Scrutiny of genome‐wide somatic mutation profiles in centenarians identifies the key genomic regions for human longevity
Source: Aging Cell. 2023 Jul 3;23(1):e13916. doi: 10.1111/acel.13916 (PMC10776117; doi:10.1111/acel.13916)
Supplement: Supplementary file 2 — Figures S1–S9 [file ACEL-23-e13916-s002.docx]

**Supplementary Figures**


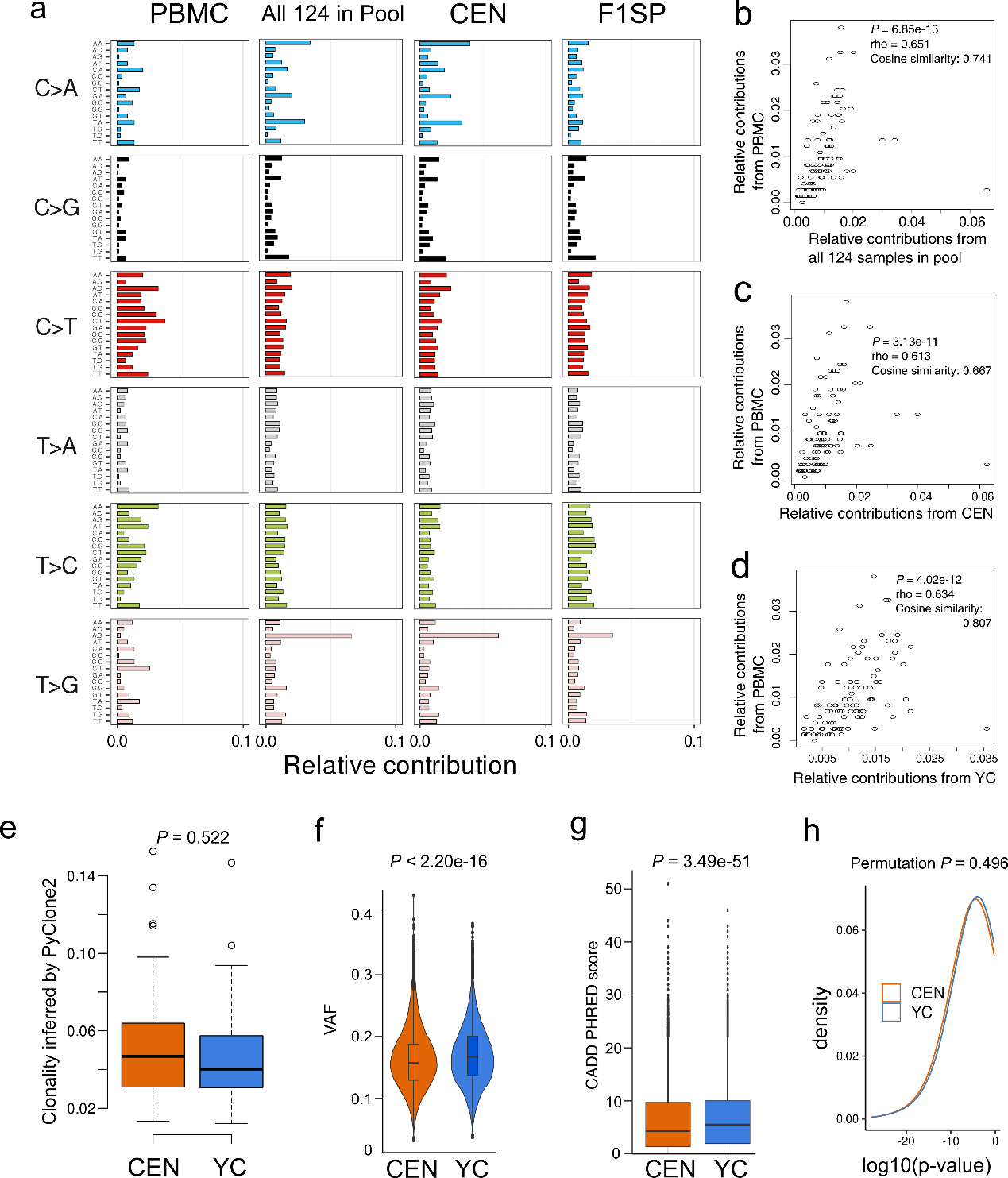


**Figure S1**. Credibility analysis for the identified somatic mutations in CENs and YCs. (**a**) The trinucleotide mutational spectra from our dataset and Xing et al. META-CS PBMC dataset. (**b-d**) The PBMC mutational spectrum was similar to those extracted from our dataset according to Spearman correlation analysis. (**e**) The estimated clonality by PyClone2. (**f**) Average VAF of CEN mutations compared with YC mutations. (**g**) CADD scores of CEN and YC mutations. (**h**) The distribution of mutation calling sensitivities in two groups. The sensitivities were determined by binomial test p-values, which represent whether the fractions of variant reads were deviated from 0.5.


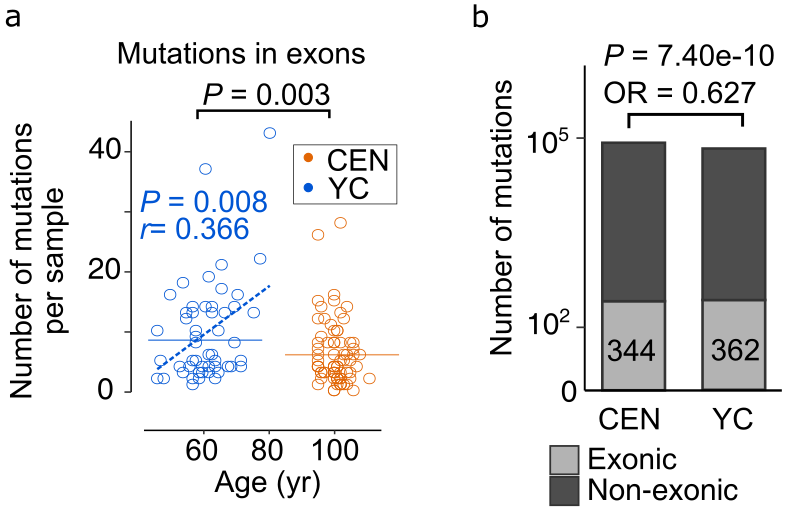


**Figure S2**. Distribution of exonic mutations in CENs and YCs. (**a**) Exonic mutation numbers in each sample. Two-sample tests were performed by Wilcoxon rank-sum test. Horizontal lines represent means. Dashed lines represent fitted lines for YC mutations. Correlations were analyzed using Pearson correlation test. Blue represents YCs and orange represents CENs. (**b**) Proportions of exonic mutations in CENs and YCs*. P*-value was calculated by Fisher’s exact test.


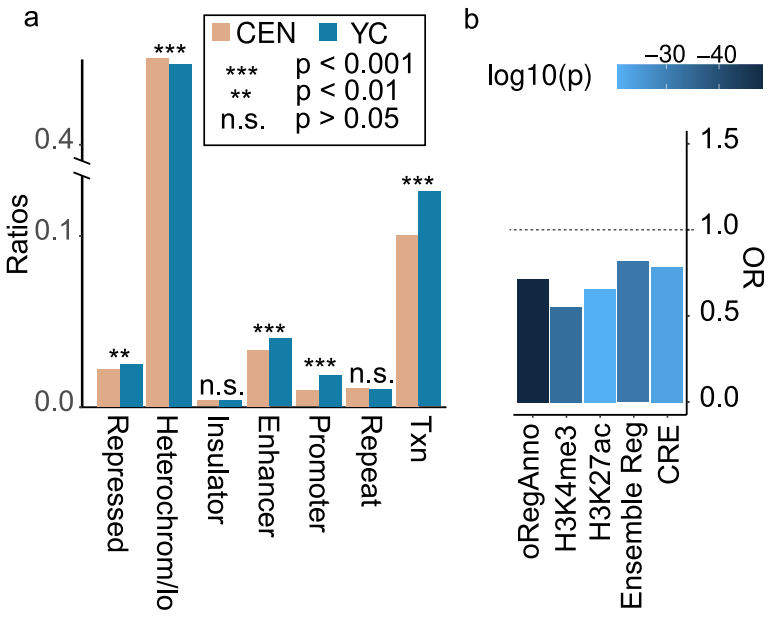


**Figure S3**. Distribution of the identified somatic mutations on different regulatory elements. (**a**) Chromatin segment distribution bias between CEN and YC mutations. *P*-values were calculated by Fisher’s exact test. (**b**) The centenarian mutations were less enriched in regulatory elements than YC’s.


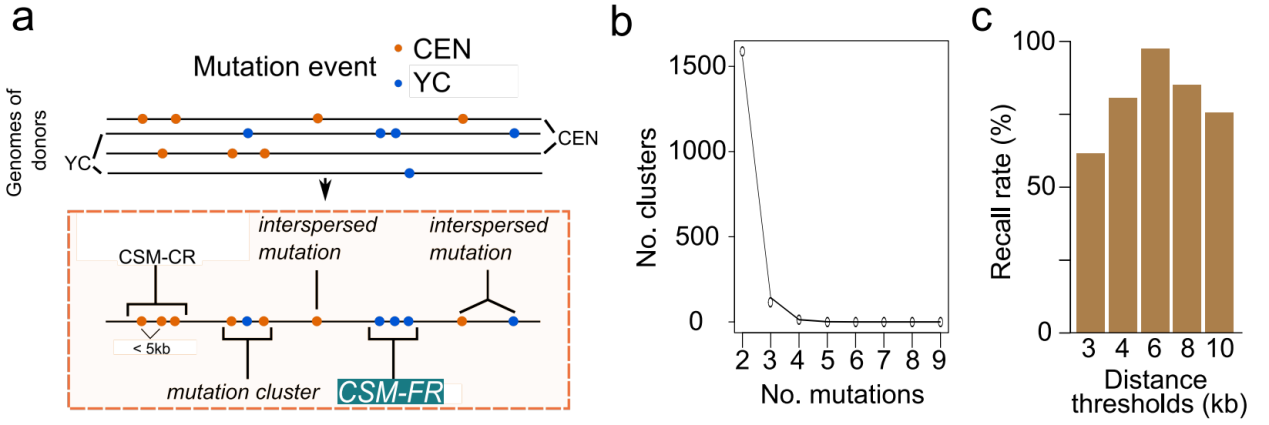


**Figure S4**. Identification of CSM-FRs. (**a**) Diagram representing pipeline of CSM-FR identification. (**b**) The somatic mutation numbers in the CSM-FRs. (**c**) The recall rates of CSM-FRs when using different distance thresholds.


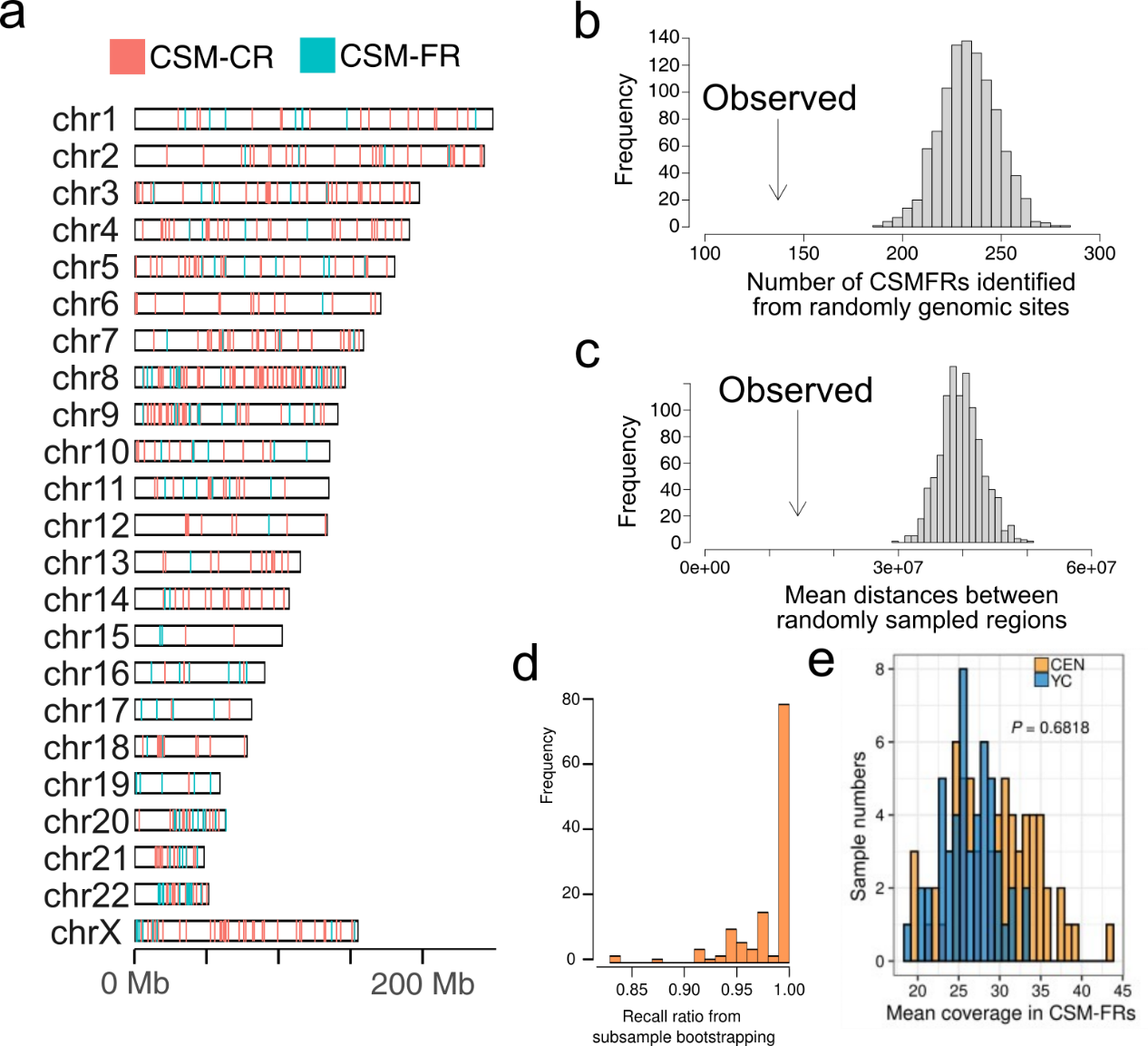


**Figure S5.** Genomic distributions of CSM-FRs and CSM-CRs. (**a**) Locations of CSM-FRs across chromosomes. (**b**) Number of CSM-FRs compared to expected number from randomly generated mutations. (**c**) Average distances between pairs of nearest CSM-FRs compared to expected distance from random genomic regions. (**d**) The recall ratios from subsamplings. (**e**) The mean coverage of CSM-FRs in both groups.


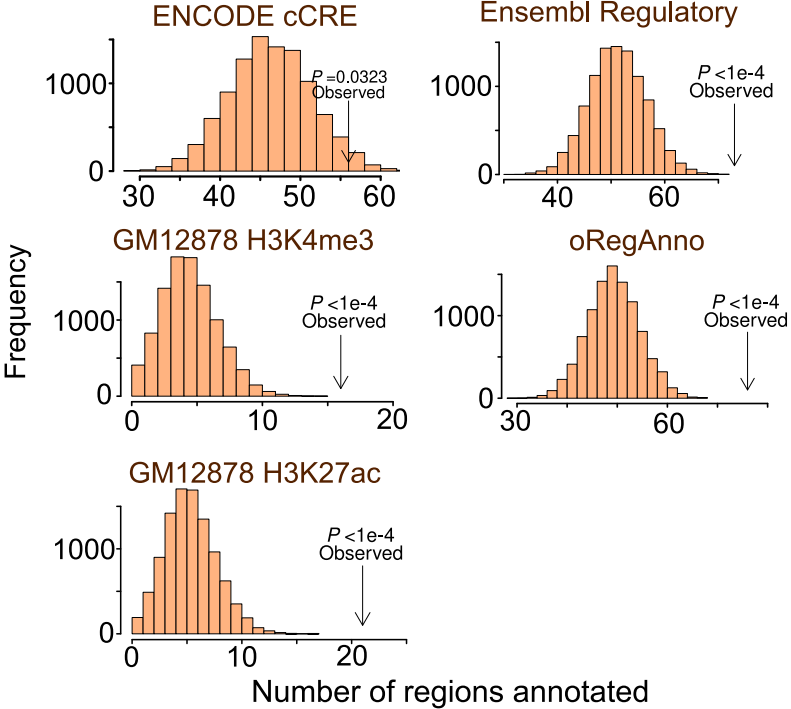


**Figure S6**. Distributions of randomly sampled regions located in regulatory elements. All *P-*values were inferred by 10 000 permutations. Arrows represent the observed numbers of the CSM-FRs located in the corresponding regulatory elements.


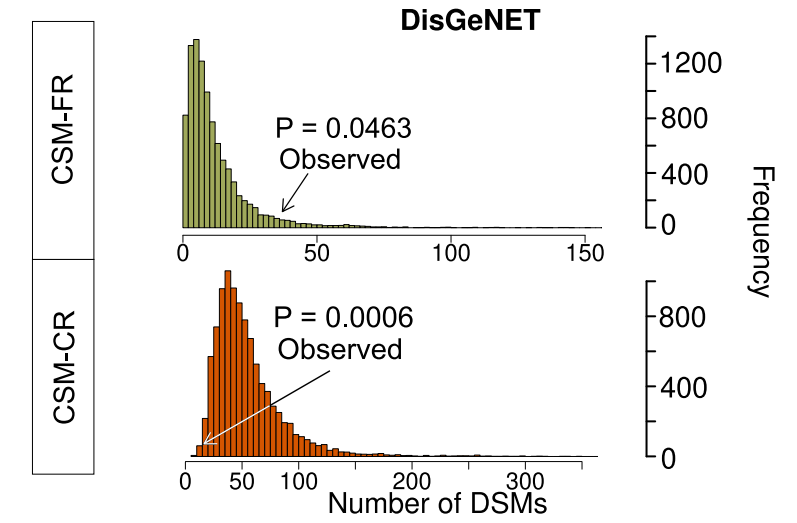


**Figure S7.** Number of DSMs located in randomly selected genomic regions. Arrows represent observed numbers in CSM-FR and CSM-CR. *P*-values were inferred by 10 000 permutations.


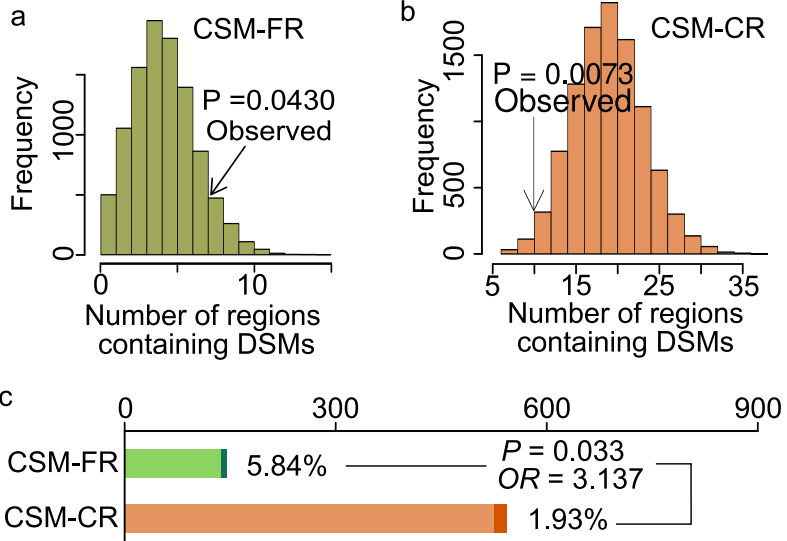


**Figure S8**. CSM-FRs preferentially sustained DSMs. (**a**) Expected distribution of random genomic regions containing DSMs. Genomic regions were generated by shuffling CSM-FRs. Arrow represents observed number. *P*-values were inferred by 10 000 permutations. (**b**) Expected distribution of random genomic regions containing DSMs. Genomic regions were generated by shuffling CSM-CRs. Arrow represents observed number. *P*-values were inferred by 10 000 permutations. (**c**) Ratio of CSM-FRs/CSM-CRs containing DSMs. *P*-value was calculated by Fisher’s exact test.


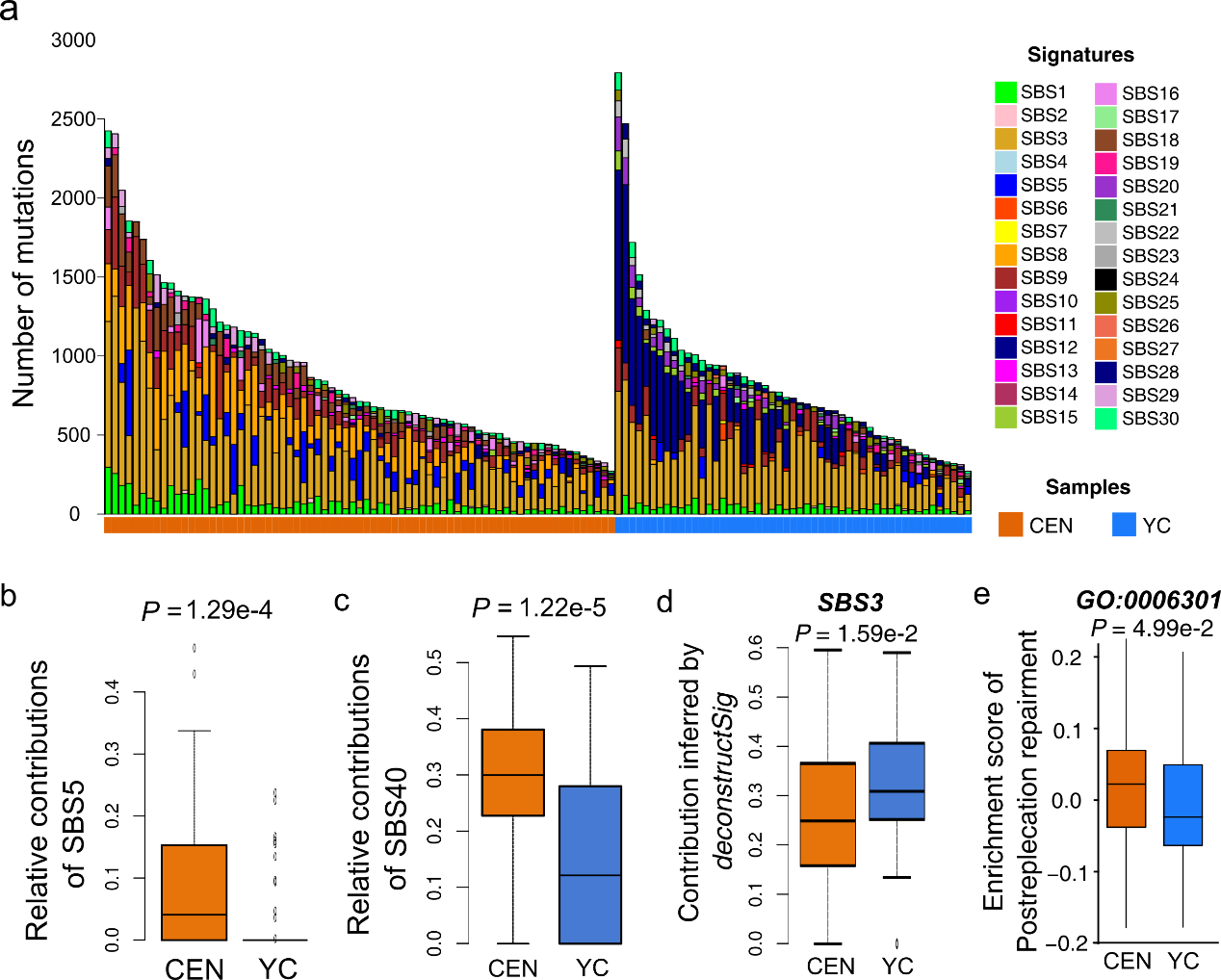


**Figure S9.** Mutational spectrum and signature analysis. (**a**) Mutational signatures extracted from each sample. (**b**-**c**) The contributions of SBS5 (**b**) and SBS40 (**c**). (**d**) Fraction of SBS3 identified by deconstructSigs. Two-sample tests were performed by Wilcoxon rank-sum test. (**e**) The pathway activity of “Postreplication repair” pathway (GO:0006301) was increased in CENs. Two-sample tests were performed by Wilcoxon rank-sum test.
